# Effect of mindfulness-based mind-body therapies in patients with non-specific low back pain—A network meta-analysis of randomized controlled trials

**DOI:** 10.3389/fnagi.2023.1148048

**Published:** 2023-06-29

**Authors:** Huanying Yang, Xiangfu Wang, Xuetao Wang, Jianxia Yang, Wanqian Zhang, Yanfang Ding, Tingrui Sang, Weiguo Chen, Wanhong Wang

**Affiliations:** ^1^Department of Traditional Chinese Orthopedics, Gansu University of Chinese Medicine, Lanzhou, Gansu, China; ^2^Department of Orthopedics, Gansu Provincial Hospital of Traditional Chinese Medicine, Lanzhou, Gansu, China; ^3^Department of Traditional Chinese Nursing, Gansu Provincial Hospital of Traditional Chinese Medicine, Lanzhou, Gansu, China

**Keywords:** mindfulness, mind-body therapies, non-operative therapy, non-specific low back pain, network meta-analysis

## Abstract

**Background/objectives:**

Although mindfulness-based mind-body therapy (MBMBT) is an effective non-surgical treatment for patients with non-specific low back pain (NLBP), the best MBMBT mode of treatment for NLBP patients has not been identified. Therefore, a network meta-analysis (NMA) was conducted to compare the effects of different MBMBTs in the treatment of NLBP patients.

**Methods:**

PubMed, EMBASE, Cochrane Central Register of Controlled Trials, and Web of Science databases were searched for randomized controlled trials (RCTs) applying MBMBT for the treatment of NLBP patients, with all of the searches ranging from the time of database creation to January 2023. After 2 researchers independently screened the literature, extracted information, and evaluated the risks of biases in the included studies, the data were analyzed by using Stata 16.0 software.

**Results:**

A total of 46 RCTs were included, including 3,886 NLBP patients and 9 MBMBT (Yoga, Ayurvedic Massage, Pilates, Craniosacral Therapy, Meditation, Meditation + Yoga, Qigong, Tai Chi, and Dance). The results of the NMA showed that Craniosacral Therapy [surface under the cumulative ranking (SUCRA): 99.2 and 99.5%] ranked the highest in terms of improving pain and disability, followed by Other Manipulations (SUCRA: 80.6 and 90.8%) and Pilates (SUCRA: 54.5 and 71.2%). In terms of improving physical health, Craniosacral Therapy (SUCRA: 100%) ranked the highest, followed by Pilates (SUCRA: 72.3%) and Meditation (SUCRA: 55.9%). In terms of improving mental health, Craniosacral Therapy (SUCRA: 100%) ranked the highest, followed by Meditation (SUCRA: 70.7%) and Pilates (SUCRA: 63.2%). However, in terms of improving pain, physical health, and mental health, Usual Care (SUCRA: 7.0, 14.2, and 11.8%, respectively) ranked lowest. Moreover, in terms of improving disability, Dance (SUCRA: 11.3%) ranked lowest.

**Conclusion:**

This NMA shows that Craniosacral Therapy may be the most effective MBMBT in treating NLBP patients and deserves to be promoted for clinical use.

**Systematic review registration:**

https://www.crd.york.ac.uk/PROSPERO/, PROSPERO [CRD42023389369].

## 1. Introduction

Low back pain is a major public health problem in modern society and one of the common symptoms in orthopedics and rehabilitation medicine. According to current surveys (Deyo and Bass, 1989), low back pain is one of the most common prevalent symptoms, and its attendance rate is second only to upper respiratory tract disorders. Globally, ~80% of the population will experience low back pain at least once in their lifetime, with a prevalence rate of 7.3%; in addition, ~6.3–15.3% of the population will develop this condition for the first time each year (Hartvigsen et al., 2018; Knezevic et al., 2021). According to surveys (Clark and Horton, 2018), people aged 40–69 years have the highest probability of suffering from low back pain, and its occurrence is gradually being observed at younger ages, with a prevalence of ~1–6% in children aged 7–10 years and ~18% in adolescents aged 11–19 years. The annual socioeconomic losses due to low back pain in the United States exceed $100 billion, with indirect losses in lost wages and decreased productivity accounting for two-thirds of these losses (Katz et al., 2006). An epidemiological survey of 195 countries worldwide showed that (James et al., 2018) years lived with disability in low back pain was the highest of all diseases, and it severely affects the physical and mental health and work capacity of patients, while also becoming the leading cause of productivity loss. In contrast, non-specific low back pain (NLBP) is the most common low back pain, accounting for ~90% of cases (Hartvigsen et al., 2018). NLBP is defined as low back pain produced by non-pathological anatomical factors other than specific low back pain (such as lumbar spinal stenosis, lumbar fracture, lumbar spine slippage, lumbar spine deformity, lumbar spine infection, malignancy, cauda equina syndrome, rheumatoid arthritis, neurogenic diseases and metabolic diseases, among other factors), which cannot be clinically determined in the etiology. The probability of recurrence in NLBP patients within 1 year is ~33% (Gatchel et al., 1995; Itz et al., 2013; da Silva et al., 2017), and NLBP has become a salient condition threatening human health.

Although there is currently no complete cure for NLBP, only some methods can be used to relieve the symptoms to a certain extent; however, the symptoms of most NLBP patients are fortunately mild and self-limited and can have a good effect through non-surgical treatment (Hlaing et al., 2021). Non-surgical treatment of NLBP mainly includes anti-inflammatory and analgesic drugs, acupuncture, traction, manual therapy, exercise and interventional therapies. Among these methods, oral anti-inflammatory and analgesic drugs are currently the most common choice to relieve NLBP; however, this method not only causes side effects such as bone loss, depression and gastric pain but also creates drug resistance with long-term use (Bishop and Wing, 2003; Maher et al., 2017). Coupled with the particularity of the disease, NLBP patients suffer from both physical pain and disability and also psychological pressure from personal, family and social aspects during the treatment period, which leads to an increased physical and mental burden on patients and seriously affects their quality of life and disease regression. Therefore, it is urgent to adopt a safe, effective, and feasible method to alleviate the symptoms and maintain the physical and mental health of NLBP patients.

There is consensus in current guidelines that the treatment of NLBP should focus on non-surgical therapy and psychosocial interventions (Bernstein et al., 2017; Qaseem et al., 2017; Stochkendahl et al., 2018). Mindfulness-based mind-body therapy (MBMBT) is an approach that emphasizes the curing of both mind and body. Mindfulness is utilized to make individuals actively aware of what they are doing at the moment, such as breathing, walking and reading, after which they can accept their feelings, look at everyone and everything around them with a new attitude, and finally return to the tranquility of the mind to find the balance between themselves and the surrounding environment (Green and Kinchen, 2021). Mind-body therapy emphasizes the interplay among the brain, mind, body, and behavior, with the intent to use the mind to affect physical functioning and to promote health (Dossett et al., 2020). Based on this connection, MBMBT utilizes mindfulness as its skeleton and specific mind-body therapies as its flesh and blood. Specifically, this concept allows individuals to listen to their own voice through current actions, thoughts, and words to change automatized behavior patterns and to heal the body's pain while enhancing the power of the mind (Fogaça et al., 2021). In recent years, the application of mindfulness-based mind-body therapy to improve the quality of life of pain patients has gradually become a research hotspot and has been widely considered and recognized by the international medical community.

Numerous studies have shown that (Okafor et al., 2012; Vohra et al., 2016; Kumar et al., 2017; Haller et al., 2019; Shi et al., 2022) Yoga, Ayurvedic Massage, Pilates, Craniosacral Therapy, Meditation, Meditation + Yoga, Qigong, Tai Chi, and Dance use mindfulness as the backbone of the therapy, both of which combine mental relaxation and physical therapy, which can effectively improve the pain and quality of life of patients with pain, as well as promote psychological wellbeing. Moreover, they are effective treatments for patients with NLBP. However, for MBMBT, different treatment modalities have different characteristics and produce different effects on NLBP patients. Lee et al. (2009) considered Qigong as an effective pain management modality; however, the difference is that (Blodt et al., 2015) observed that other exercise therapies reduced pain and dysfunction more than Qigong in 114 NLBP patients. Compared with Qigong, Yoga is also an easy-to-implement MBMBT. Nambi et al. (2014) demonstrated that Yoga better improved pain than other exercises in NLBP patients through a 12-month randomized controlled trial. Based on the trials of Nambi et al. (2014) and Blodt et al. (2015), Yoga should be superior to Qigong in the treatment of NLBP patients. However, Teut et al. (2016) directly compared the effectiveness of pranayama and Yoga in treating NLBP patients and found that pranayama was superior to Yoga in improving pain and mental health.

Although a large number of clinical trials have confirmed the advantages of MBMBT in treating NLBP patients, the findings of different studies have exhibited significant differences (de Freitas et al., 2020). To date, no research has conducted a systematic evidence-based medical study on this topic. Therefore, there is an immediate need to identify an optimal modality in MBMBT to improve the symptoms associated with NLBP patients. A network meta-analysis (NMA) can subsequently directly and indirectly compare the efficacy of multiple interventions, synthesize the pros and cons of multiple interventions, and rank the effectiveness of multiple treatments to select the most suitable clinical treatment option for the patient. Based on this scenario, this paper performed an NMA on randomized controlled trials (RCTs) of different MBMBT (Yoga, Ayurvedic Massage, Pilates, Craniosacral Therapy, Meditation, Meditation + Yoga, Qigong, Tai Chi, and Dance) for NLBP patients to compare the effectiveness of nine approaches in improving pain, disability, and the physical and mental health of NLBP patients and to select the optimal MBMBT, which contributes to a better understanding of the effectiveness of MBMBT for patients and clinicians.

## 2. Materials and methods

This NMA has been successfully registered with PROSPERO (ID: CRD42023389369) and was performed in strict compliance with the Cochrane Handbook (Cumpston et al., 2019) and the PRISMA-NMA statement (Hutton et al., 2015).

### 2.1. Search strategy

The researchers of this review searched the PubMed, EMBASE, Cochrane Central Register of Controlled Trials, and Web of Science databases for relevant literature from inception to January 2023 to identify RCTs of MBMBT for NLBP patients. The full search strategy is available in Table 1 (using PubMed as an example). Additionally, lists of references of relevant systematic reviews and meta-analyses were checked.

**Table 1:** Search strategy on PubMed.

**#1**	**(((nonspecific low back pain) OR (non-specific low back pain)) OR (NSLBP)) OR (NLBP)**
#2	Mind-Body Therapies[Mesh]
#3	(((((((((((((((((((((biofeedback, psychology) OR (beurofeedback)) OR (breathing exercises)) OR (qigong)) OR (aromatherapy)) OR (laughter therapy)) OR ((imagery, psychotherapy)) OR (meditation)) OR (mental Healing)) OR (psychodrama)) OR (role playing)) OR (hypnosis)) OR (autogenic training)) OR (suggestion)) OR (psychophysiology)) OR (tai chi)) OR (relaxation therapy)) OR (therapeutic touch)) OR (yoga)) OR (dance therapy)) OR (pilates)) OR (baduanjin)
#4	#2 OR #3
#5	#1 AND #4

### 2.2. Inclusion criteria

The inclusion criteria were framed according to the PICOS strategy. (P) Population: people over 18 years of age with NLBP; (I) Intervention: different MBMBT as a categorized intervention (Yoga, Ayurvedic Massage, Pilates, Craniosacral Therapy, Meditation, Qigong, Tai Chi or Dance); (C) Comparator: control participants with only usual care and appropriate rehabilitation measures being used; (O) Outcomes: self-reported outcomes in people with NLBP (including at least one of the following outcomes of interest: pain, disability, physical health or mental health); and (S) Study type: RCTs.

### 2.3. Exclusion criteria

The following exclusion criteria were used. (1) Studies with duplicate publications; (2) studies with incomplete data; and (3) studies from non-RCTs (including protocols, animal studies, conference abstracts, correspondences, case reports, or quasirandomized controlled trials).

### 2.4. Study selection

All of the search results were managed by using the literature management software Endnote 20. First, duplicates were excluded by using Endnote 20, after which Ding and Sang read the titles and abstracts of the literature to independently perform an initial screening of the literature based on the inclusion and exclusion criteria. Afterwards, they further read the full text for rescreening to determine inclusion or not. Finally, Wang synthesized the remaining literature and adjudicated all of the disagreements.

### 2.5. Data extraction

In this study, the data extraction were classified into the following six categories: (1) author's name, (2) year of publication, (3) place, (4) sample features (size, age, and sex), (5) intervention measures, and (6) outcomes. All of the abovementioned data extractions were independently completed by Ding and Chen, and all disagreements were adjudicated by Wang.

Although the assessment methods in each extracted outcome measure in this NMA are not consistent, they all belong to the most commonly used and recognized methods of NLBP assessment in the medical community. Pain measures were extracted from the results of the visual analog scale (VAS), Numerical Pain Intensity Scale (NPRS), McGill Pain Questionnaire (MPQ), and brief pain inventory (BPI). In addition, disability measures were extracted from the results of the Oswestry Disability Index (ODI), Quebec Back Pain Disability Scale (QBPDS), and Roland-Morris Disability Questionnaire (RMDQ). Both physical and mental health measures were extracted from the results of the SF-36 and SF-12.

### 2.6. Risk of bias of individual studies

Sang and Chen independently evaluated the risk of bias in the included literature in this study according to the Cochrane Risk of Bias Tool in the Cochrane handbook (Sterne et al., 2019). The following seven domains of risk of bias were analyzed: (1) randomized sequence generation, (2) allocation concealment, (3) blinding of participants and personnel, (4) blinding of outcome assessment, (5) incomplete outcome data, (6) selective outcome reporting, and (7) other sources of bias. The risk of bias in each domain was categorized into three levels: low risk, high risk, and unclear risk. All disagreements were adjudicated by Wang.

### 2.7. Data analysis

In all of the included literature, variables were continuous, and the extracted outcome data included the postintervention mean and standard deviation, which were converted to means and standard deviations before inclusion when the data were expressed in other forms in the literature. Standardized mean differences (SMDs) and their 95% confidence intervals (CIs) were calculated and analyzed for all of the extracted data. Accounting for the potential differences between different types of literature, this NMA used a random-effects model for analysis rather than a fixed-effects model.

First, a network map of direct comparisons between different interventions was drawn by using Stata software (version 16.0). Each node in the map represents an intervention, and the size of the node indicates the sample size receiving the intervention. The presence of a line between two nodes indicates that they have a direct comparison relationship, and a thicker line indicates a higher number of comparisons. Subsequently, the agreement between direct and indirect comparisons was assessed via the node-splitting method and was considered good if *P* > 0.05.

Additionally, the effects of various interventions were quantitatively analyzed by using the surface under the cumulative ranking (SUCRA) to rank the effects of different interventions. SUCRA values range from 0 to 100%, and if the SUCRA value for an intervention is closer to 100%, it indicates that the intervention is more effective. However, this conclusion should be interpreted with caution if there is not a clinically meaningful difference between the two interventions. Finally, network funnel plots were drawn and visually checked by using the symmetry criterion to determine if there was a possibility of bias leading to NMA publication.

## 3. Results

### 3.1. Study and identification and selection

**Figure 1:**
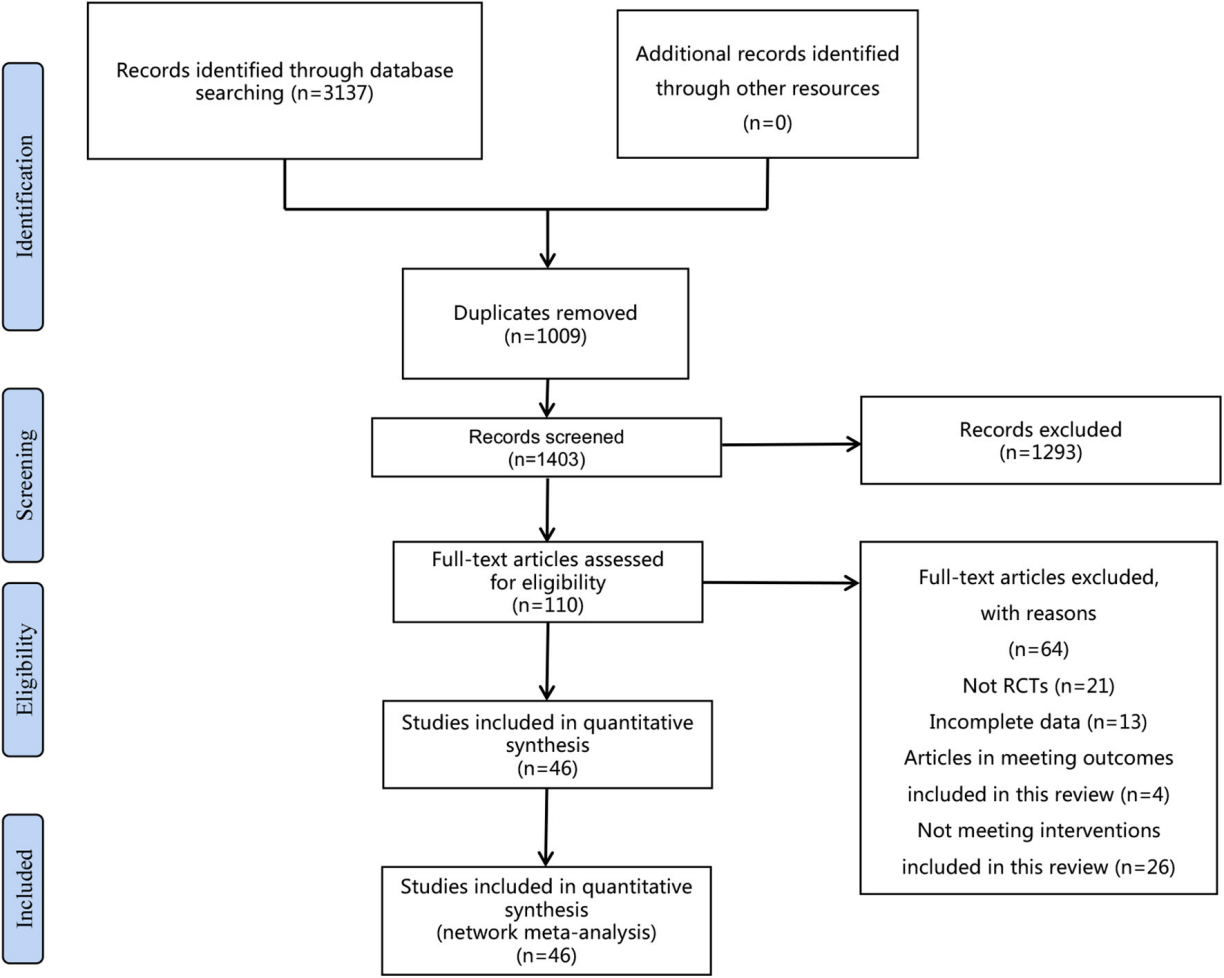
Flow diagram of literature selection.

A total of 3,137 relevant types of literature were obtained from the initial search of the four databases. In the initial search results, 1,009 duplicates were first excluded by using Endnote 20. A total of 1,293 articles were eliminated by the initial reading of the titles and abstracts. Subsequently, a further full-text analysis of the literature according to inclusion and exclusion criteria excluded 64 studies, including non-RCTs (*n* = 21), incomplete data (*n* = 13), articles in meeting outcomes that were included in this review (*n* = 4), and non-meeting interventions included in this review (*n* = 26). The final 46 pieces of literature were obtained (Figure 1). Detailed search results for different databases are shown in Supplementary Table 1.

### 3.2. Quality assessment of the included studies

**Figure 2:**
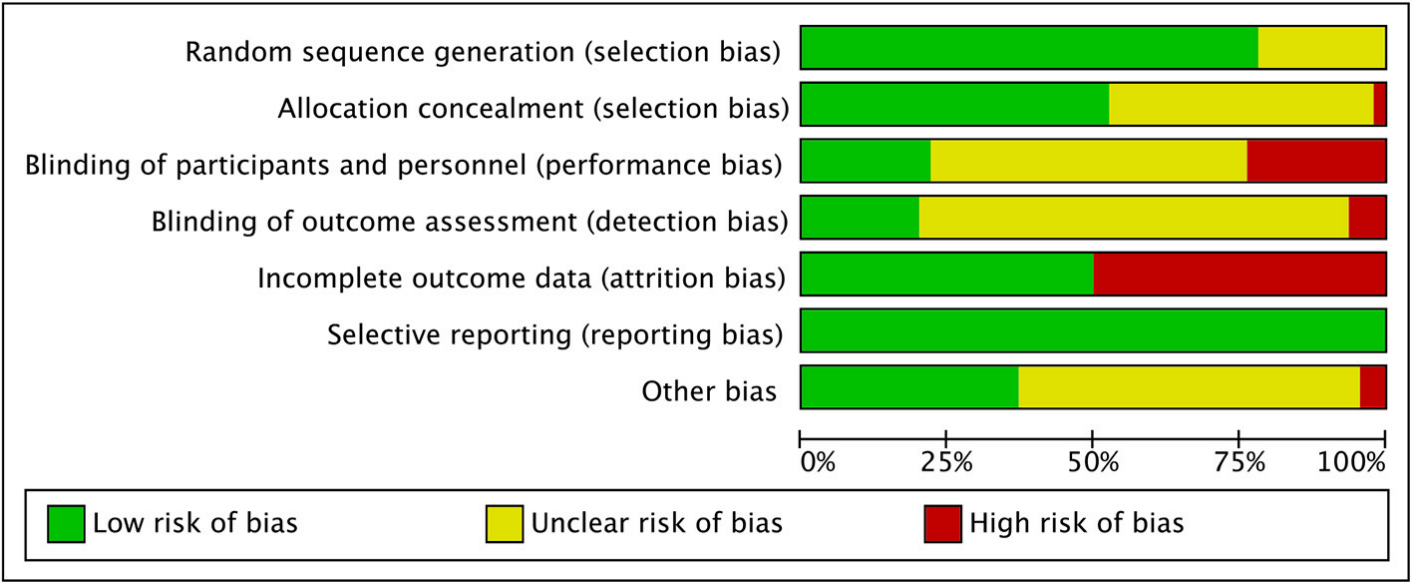
Risk of bias graph of all literature.

**Figure 3:**
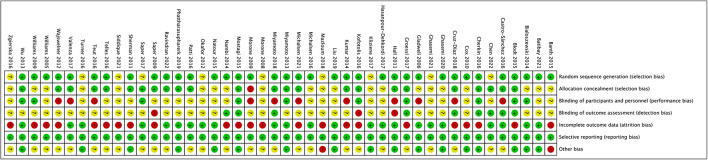
Risk of bias summary of all literature.

The quality assessment of the included studies is shown in Figures 2, 3. Thirty-six studies reported of specific random sequence generation methods, and 10 studies did not specify specific random sequence generation methods. Twenty-four studies reported of specific allocation concealment methods, one study in which the principal investigator was involved in random allocation, and 21 studies in which the specific allocation concealment method was unclear. In addition, 10 studies were blinded to participants and study personnel, nine studies were not blinded to study personnel, two studies were not blinded to participants, and there were 25 studies in which participants and researchers were not specifically blinded. Nine studies blinded outcome raters, three studies did not blind outcome raters, and 34 studies did not specify whether outcome raters were blinded. Moreover, 21 studies did not report incomplete outcome data, and 25 publications were lost to follow-up; however, only two studies provided a clear explanation of the specific reasons for loss to follow-up. Selectivity was not reported in any literature.

### 3.3. Characteristics of the included studies

A total of 46 RCTs involving 3,886 NLBP patients were included in this review. The interventions in the trial group included Yoga (Williams et al., 2005, 2009; Saper et al., 2009, 2017; Cox et al., 2010; Sherman et al., 2011; Nambi et al., 2014; Telles et al., 2016; Groessl et al., 2017; Michalsen et al., 2021) (10 studies), Ayurvedic massage (Kumar et al., 2017) (one study), Pilates (Gladwell et al., 2006; Wajswelner et al., 2012; Miyamoto et al., 2013, 2018; Mostagi et al., 2015; Natour et al., 2015; Kofotolis et al., 2016; Patti et al., 2016; Hasanpour-Dehkordi et al., 2017; Kliziene et al., 2017; Valenza et al., 2017; Cruz-Díaz et al., 2018; Mazloum et al., 2018; Batibay et al., 2021; Siddique et al., 2021; Ravindran et al., 2022) (16 studies), cranio-sacral therapy (Bialoszewski et al., 2014; Castro-Sánchez et al., 2016; Ghasemi et al., 2020, 2021) (four studies), Meditation (Morone et al., 2008, 2009; Banth and Ardebil, 2015; Michalsen et al., 2016; Zgierska et al., 2016) (five studies), Meditation + Yoga (Cherkin et al., 2016; Turner et al., 2016; Chen et al., 2023) (three studies), Qigong (Blodt et al., 2015; Teut et al., 2016; Phattharasupharerk et al., 2019) (three studies), Tai Chi (Hall et al., 2011; Weifen et al., 2013; Liu et al., 2019) (three studies), and Dance (Okafor et al., 2012) (one study). There were three control groups to compare (including Usual Care, Other Exercises, and Other Manipulation). The characteristics of the included literature are shown in Table 2.

**Table 2:** Characteristics of the studies included in the NMA.

**Authors**	**Country**	**Year**	**Population**	**Age (mean + SD)**	**Total/male/ female**	**Intervention**	**Control**	**Outcome**
Saper	USA	2017	NLBP > 12 weeks	T: 46.4 (10.4) C: 44.2 (10.8)	T: 127/55/72 C: 64/22/42	Yoga Length of Intervention: 12 weeks Freq: 1 times a week Duration: 75 min	CON	Pain, disability, physical health, mental health
Telles	India	2016	NA	T: 36.1 (7.33) C: 37.4 (4.85)	T: 20/9/11 C: 20/8/12	Yoga Length of Intervention: 12 weeks Freq: 7 times a week Duration: 60 min	CON	Pain
Williams	USA	2005	NLBP > 12 weeks	T: 48.7 (10.6) C: 48 (1.96)	T: 20/7/13 C: 24/7/17	Yoga Length of Intervention: 16 weeks Freq: 1 times a week Duration: 90 min	CON	Pain, disability
Nambi	India	2014	NLBP > 12 weeks	T: 44.26 (9.26) C: 43.66 (8.82)	T: 30/11/19 C: 30/17/13	Yoga Length of Intervention: 4 weeks Freq: 1 times a week Duration: 60 min	OE	Pain
Williams	USA	2009	NLBP > 12 weeks	T: 48.4 (1.86) C: 47.6 (1.47)	T: 43/11/32 C: 47/10/37	Yoga Length of intervention: 24 weeks Freq: 2 times a week Duration: 90 min	CON	Pain, disability
Saper	USA	2009	NLBP > 12 weeks	T: 44 (13) C: 44 (11)	T: 15/4/11 C: 15/1/14	Yoga Length of intervention: 12 weeks Freq: 1 times a week Duration: 75 min	CON	Pain, disability
Michalsen	Germany	2021	NLBP > 12 weeks	T: 53.9 (10.7) C1: 56 (9.8) C2: 53.9 (13.4)	T: 100/36/64 C1: 92/23/69 C2: 82/28/54	Yoga Length of intervention: 8 weeks Freq: 1 times a week Duration: 75 min	C1: OE C2: CON	Pain, disability, physical health, mental health
Cox	USA	2010	NLBP > 72 weeks	NA	T: 10/2/8 C: 10/5/5	Yoga Length of intervention: 12 weeks Freq: 1 times a week Duration: 75 min	CON	Pain, disability, physical health, mental health
Groessl	USA	2017	NLBP > 24 weeks	T: 53.5 (12.7) C: 53.6 (13.9)	T: 75/NA/NA C: 12/NA/NA	Yoga Length of intervention: 12 weeks Freq: 2 times a week Duration: 60 min	CON	Pain, disability
Sherman	USA	2011	NLBP > 12 weeks	T: 46.6 (9.8) C: 49 (9.91)	T: 92/30/62 C: 91/34/57	Yoga Length of intervention: 12 weeks Freq: 1 times a week Duration: 75 min	C: OE	Disability
Kumar	Germany	2014	NLBP > 12 weeks	T: 55.4 (11.2) C: 54.2 (13.8)	T: 32/6/26 C: 32/9/23	Ayurvedic massage Length of intervention: 2 weeks Freq: 3 times a week Duration: 35 min	CON	Pain, disability, physical health, mental health
Gladwell	UK	2006	NLBP > 12 weeks	T: 36.9 (8.1) C: 45.9 (8)	T: 20/4/10 C: 14/3/11	Pilates Length of intervention: 6 weeks Freq: 1 times a week Duration: 60 min	CON	Pain, disability
Batibay	Turkey	2021	NLBP > 12 weeks	T: 49.3 (10.4) C: 48.4 (9.3)	T: 28/NA/NA C: 25/NA/NA	Pilates Length of intervention: 8 weeks Freq: 3 times a week Duration: 60 min	OE	Pain, disability, physical health,
Mostagi	Brazil	2015	NLBP > 24 weeks	T: 36.1 (9) C: 34.7 (8.1)	T: 11/2/9 C: 11/2/9	Pilates Length of intervention: 8 weeks Freq: 2 times a week Duration: NA	OE	Pain, disability
Cruz-Díaz	Spain	2018	NLBP > 12 weeks	T: 37.9 (8.2) C: 35.6 (6.7)	T: 32/11/21 C: 30/10/20	Pilates Length of intervention: 12 weeks Freq: 2 times a week Duration: 50 min	CON	Pain, disability
Ravindran	India	2022	NA	T: 53 (12) C: 52.5 (8.75)	T: 23/0/23 C: 24/0/24	Pilates Length of intervention: 4 weeks Freq: 3 times a week Duration: 40 min	OE	Pain, disability
Siddique	Pakistan	2021	NA	T: 48.4 (6.14) C: 50 (8.69)	T: 13/NA/NA C: 13/NA/NA	Pilates Length of intervention: 4 weeks Freq: 3 times a week Duration: 30 min	OE	Pain, disability
Natour	Brazil	2015	NLBP > 12 weeks	T: 47.79 (11.47) C: 48.08 (12.98)	T: 30/6/24 C: 30/7/23	Pilates Length of intervention: 12 weeks Freq: 2 times a week Duration: 50 min	CON	Pain, disability, physical health, mental health
Miyamoto	Brazil	2013	NLBP > 12 weeks	T: 40.7 (11.8) C: 38.3 (11.4)	T: 43/NA/NA C: 43/NA/NA	Pilates Length of intervention: 6 weeks Freq: 2 times a week Duration: 60 min	CON	Pain, disability
Miyamoto	Brazil	2018	NLBP > 12 weeks	T: 48.9 (16.6) C: 48.6 (15.8)	T: 74/NA/NA C: 74/NA/NA	Pilates Length of intervention: 6 weeks Freq: 3 times a week Duration: 60 min	CON	Pain, disability
Kliziene	Lithuania	2017	NLBP > 12 weeks	T: 45.31 (4.31) C: 46.25 (3.26)	T: 27/0/27 C: 27/0/27	Pilates Length of intervention: 16 weeks Freq: 2 times a week Duration: 60 min	CON	Pain
Patti	Italy	2016	NLBP > 12 weeks	T: 43.31 (11.24) C: 41.63 (13.01)	T: 19/NA/NA C: 19/NA/NA	Pilates Length of intervention: 14 weeks Freq: 3 times a week Duration: 50 min	CON	Pain, disability
Valenza	Spain	2017	NLBP > 12 weeks	T: 40 (16) C: 38 (12)	T: 27/7/20 C: 27/5/22	Pilates Length of intervention: 8 weeks Freq: 2 times a week Duration: 45 min	CON	Pain, disability
Hasanpour-Dehkordi	Iran	2017	NLBP > 12 weeks	T: NA C1: NA C2: NA	T: 12/NA/NA C1: 12/NA/NA C2: 12/NA/NA	Pilates Length of intervention: 6 weeks Freq: 3 times a week Duration: 60 min	C1:OE C2:CON	Pain
Kofotolis	Greece	2016	NLBP > 12 weeks	T: 41.22 (8.49) C1: 39.11 (8.68) C2: 42.71 (6.1)	T: 40/NA/NA C1: 40/NA/NA C2: 40/NA/NA	Pilates Length of intervention: 8 weeks Freq: 3 times a week Duration: 60 min	C1:OE C2:CON	Disability, physical health, mental health
Mazloum	Iran	2018	NLBP > 12 weeks	T: 37.1 (9.5) C: 42.7 (8.1)	T: 16/NA/NA C: 15/NA/NA	Pilates Length of intervention: 6 weeks Freq: 3 times a week Duration: NA	OE	Pain, disability
Wajswelner	Australia	2012	NLBP > 12 weeks	T: 49.3 (14.1) C: 48.9 (16.4)	T: 44/19/25 C: 43/20/23	Pilates Length of intervention: 6 weeks Freq: 2 times a week Duration: 60 min	OE	Pain, physical health, mental health
Bialoszewski	Polska	2014	NA	T: 33 (6) C: 33 (7)	T: 27/NA/NA C: 28/NA/NA	Craniosacral therapy Length of intervention: 3–4 day Freq: took part in three sessions at 3–4 day intervals Duration: NA	OM	Pain
Ghasemi	Iran	2021	NLBP > 24 weeks	T: 27.7 (4.8) C: 27.4 (3.5)	T: 16/8/8 C: 15/9/6	Craniosacral therapy Length of intervention: 5 weeks Freq: 2 times a week Duration: 45 min	OE	Pain, disability, physical health, mental health
Ghasemi	Iran	2020	NLBP > 12 weeks	T: NA C1: NA C2: NA	T: 15/NA/NA C1: 15/NA/NA C2: 15/NA/NA	Craniosacral therapy Length of intervention: 5 weeks Freq: 2 times a week Duration: 45 min	C1:OE C2:OM	Pain, disability, physical health, mental health
Castro-Sánchez	Spain	2016	NLBP > 12 weeks	T: 50 (11) C: 53 (9)	T: 32/10/23 C: 32/12/19	Craniosacral Therapy Length of intervention: 10 weeks Freq: 1 times a week Duration: 50 min	OM	Pain, disability
Banth	India	2015	NLBP > 24 weeks	T + C: 40.3 (8.2)	T: 39/0/39 C: 48/0/48	Meditation Length of intervention: 8 weeks Freq: 1 times a week Duration: 90 min	CON	Pain, physical health, mental health
Morone	USA	2008	NLBP > 12 weeks	T: 74.1 (6.1) C: 75.6 (5)	T: 19/9/10 C: 18/7/11	Meditation Length of intervention: 8 weeks Freq: 8 times a week Duration: 90 min	CON	Pain, disability, physical health, mental health
Morone	UAS	2009	NLBP > 12 weeks	T: 78 (7.1) C: 73 (6.2)	T: 16/5/11 C: 19/8/11	Meditation Length of intervention: 8 weeks Freq: 1 times a week Duration: 90 min	CON	Pain, disability
Michalsen	Germany	2016	NLBP > 12 weeks	T: 55.5 (10.6) C: 54.8 (10.6)	T: 32/3/29 C: 36/13/23	Meditation Length of intervention: 8 weeks Freq: NA Duration: a weekly 90 min	OE	Pain, disability, physical health, mental health
Zgierska	USA	2016	NLBP > 12 weeks	T + C: 51.8 (9.7)	T: 21/NA/NA C: 14/NA/NA	Meditation Length of intervention: 8 weeks Freq: NA Duration: 2 h per week	CON	Pain, disability
Turner	USA	2016	NLBP > 12 weeks	T: 50 (11.9) C: 48.9 (12.5)	T: 116/45/71 C: 113/26/87	Meditation + Yoga Length of intervention: 8 weeks Freq: NA Duration: 2 h per week	CON + Yoga	Pain
Cherkin	USA	2016	NLBP > 12 weeks	T: 50 (11.9) C: 48.9 (12.5)	T: 116/45/71 C: 113/26/87	Meditation + Yoga Length of intervention: 8 weeks Freq: NA Duration: 2 h a week	CON	Pain, disability
Chen	USA	2022	NLBP > 12 weeks	T: NA C: NA	T: 94/36/58 C: 106/24/82	Meditation + Yoga Length of intervention: 8 weeks Freq: NA Duration: weekly 2-hour	CON	Pain, disability
Phatthara- supharerk	Thailand	2019	NLBP > 12 weeks	T: 35.7 (3.6) C: 34.8 (4.3)	T: 36/12/24 C: 36/14/22	Qigong Length of intervention: 6 weeks Freq: NA Duration: 1 h per week	CON	Pain, disability
Teut	Germany	2016	NLBP > 24 weeks	T: 72.4 (5.7) C: 73 (5.6)	T: 58/8/50 C: 61/7/54	Qigong Length of intervention: 12 weeks Freq: 12 times over 3 month Duration: 90 min	Yoga	Pain, disability, physical health, mental health
Blodt	Germany	2015	NLBP > 12 weeks	T: 45.7 (10) C: 47.7 (10.8)	T: 64/6/58 C: 63/19/44	Qigong Length of intervention: 12 weeks Freq: 1 times a week Duration: 90 min	OE	Pain, disability, physical health, mental health
Liu	China	2019	NLBP > 12 weeks	T: 58.13 (5.38) C1: 58.4 (5.08) C2: 60.67 (2.58)	T: 15/4/11 C1: 15/4/11 C2: 13/3/10	Tai Chi Length of intervention: 12 weeks Freq: 3 times a week Duration: 60 min	C1:OE C2:CON	Pain
Wu	China	2013	NLBP 48–240 weeks	T: 37.5 (5.2) C: 37.5 (5.5)	T: 141/86/55 C: 38/21/17	Tai Chi Length of intervention: 24 weeks Freq: 5 times a week Duration: 45 min	OE	Pain
Hall	Australia	2011	NA	T: 43.4 (13.5) C: 44.3(13)	T: 80/17/63 C: 80/24/56	Tai Chi Length of intervention: 18 weeks Freq: 2 times per week for 8 weeks followed by once per week for 2 weeks Duration: 40 min	CON	Pain, disability
Okafor	Nigeria	2012	NLBP	T: NA C: NA	T: 15/5/10 C: 15/5/10	Dance Length of intervention: 6 weeks Freq: 3 times a week Duration: 45 min	CON	Pain, disability

CON, control group with usual care; OE, control group with other exercise; OM, control group with other manipulation; T, experimental group; C, control group; TRD, treadmill training; T + C, The ages of the experimental and control groups were not reported separately in the study, only the overall age was reported; NA, unavailable; Freq, frequency.

### 3.4. NMA

**Figure 4:**
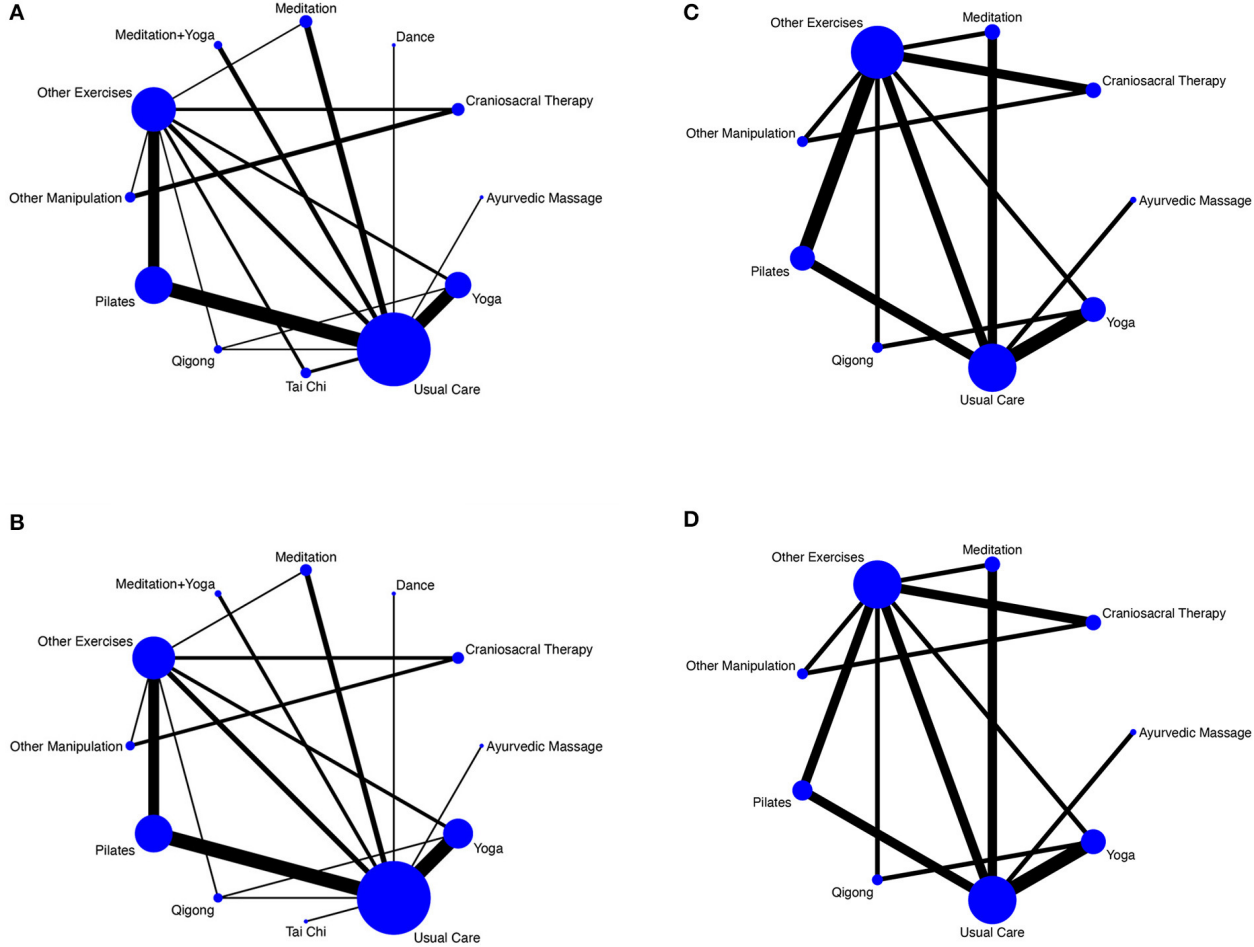
**(A)** NMA figure for pain. **(B)** NMA figure for disability. **(C)** NMA figure for physical health. **(D)** NMA figure for mental health.

The NMA map is shown in Figures 4A–D.

#### 3.4.1. Pain

**Figure 5:**
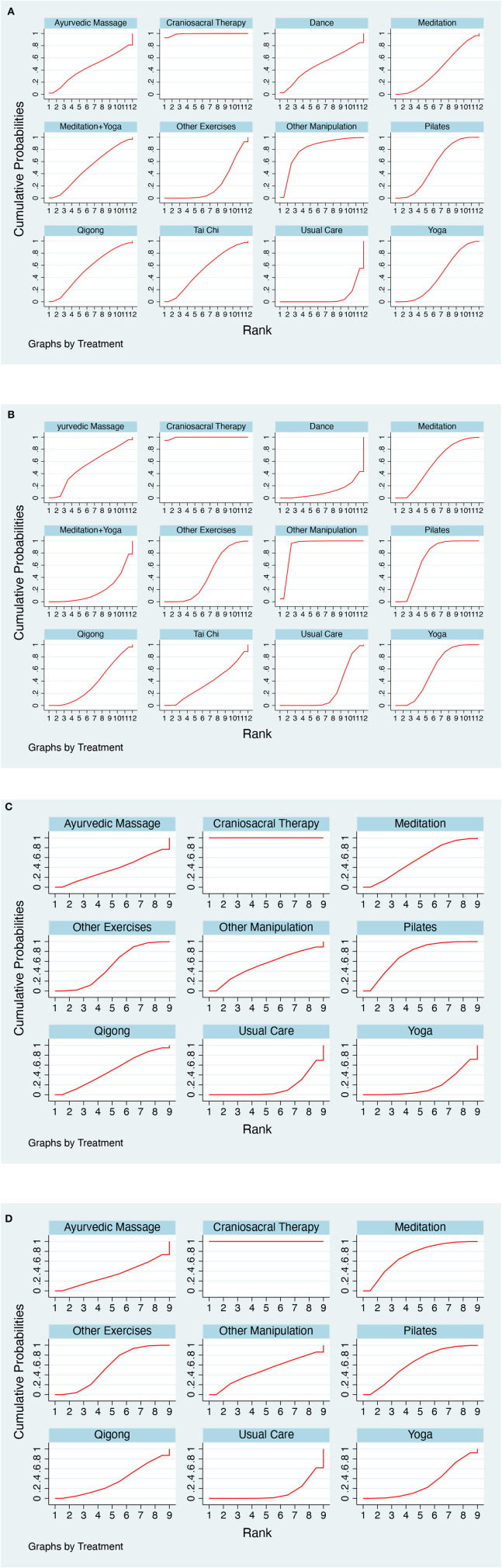
**(A)** SUCRA plot for pain. **(B)** SUCRA plot for disability. **(C)** SUCRA plot for physical health. **(D)** SUCRA plot for mental health.

A total of 44 studies reported of pain, including 12 interventions and 3,411 participants. The results of the NMA showed that compared with Usual Care, Craniosacral Therapy [SMD = −4.59, 95% CI = (−6.76 to −2.42)], Other Manipulation [SMD = −2.74, 95% CI = (−5.06 to −0.42)], Pilates [SMD = −1.30, 95% CI = (−2.08 to −0.51)], and Yoga [SMD = −1.02, 95% CI = (−1.89 to −0.14)] were all superior to Usual Care in improving pain. Details are shown in Table 3A. There was no significant inconsistency in the results of the node-splitting method for most of the comparisons (*P* > 0.05), thus suggesting good agreement between direct and indirect comparisons for most of the studies. The details of this analysis are shown in Supplementary Table 2A. The results of the SUCRA ranking of different interventions in terms of improving pain were Craniosacral Therapy (99.2%) > Other Manipulations (80.6%) > Pilates (54.5%) > Qigong (54.1%) > Tai Chi (53.7%) > Meditation + Yoga (50.6%) > Dance (48.6%) > Ayurvedic Massage (44.2%) > Yoga (43.1%) > Meditation (42.1%) > Other Exercises (22.4%) > Usual Care (7.0%). The details of this ranking are shown in Figure 5A.

**Table 3A:** League table on pain and disability.

	**Disability**												
**Pain**	**Craniosacral Therapy**	1.13 (−0.21, 2.47)	**3.34 (1.69, 5.00)**	**4.20 (2.28, 6.12)**	**4.26 (1.75, 6.76)**	**4.81 (2.68, 6.93)**	**5.38 (2.78, 7.97)**	**3.68 (1.12, 6.23)**	**3.70 (1.96, 5.43)**	**3.70 (1.82, 5.58)**	**4.01 (2.50, 5.53)**	**4.47 (2.81, 6.13)**
		**−1.85 (−3.39,−0.30)**	**Other manipulation**	2.21 (0.39, 4.03)	**3.07 (1.00, 5.14)**	3.13 (0.51, 5.74)	**3.67 (1.42, 5.93)**	**4.24 (1.54, 6.95)**	2.54 (−0.12, 5.21)	**2.56 (0.67, 4.46)**	2.57 (0.54, 4.59)	**2.88 (1.19, 4.57)**	**3.34 (1.51, 5.17)**
		**−3.29 (−5.45,−1.14)**	−1.45 (−3.75, 0.86)	**Pilates**	0.86 (−0.38, 2.09)	0.91 (−1.05, 2.88)	1.46 (0.01, 2.92)	2.03 (−0.05, 4.12)	0.33 (−1.70, 2.36)	0.35 (−0.49, 1.20)	0.36 (−0.78, 1.49)	0.67 (−0.01, 1.35)	1.13 (0.52, 1.74)
		**−3.21 (−5.79,−0.64)**	−1.37 (−4.07, 1.33)	0.08 (−1.63, 1.78)	**Qigong**	0.06 (−2.14, 2.25)	0.61 (−1.14, 2.35)	1.17 (−1.13, 3.47)	−0.53 (−2.78, 1.73)	−0.51 (−1.68, 0.67)	−0.50 (−1.99, 0.99)	−0.19 (−1.37, 1.00)	0.27 (−0.88, 1.41)
		**−3.26 (−5.76,−0.76)**	−1.42 (−4.05, 1.22)	0.03 (−1.59, 1.66)	−0.05 (−2.19, 2.10)	**Tai Chi**	0.55 (−1.74, 2.84)	1.12 (−1.62, 3.85)	−0.58 (−3.27, 2.11)	−0.56 (−2.54, 1.42)	−0.56 (−2.68, 1.57)	−0.24 (−2.24, 1.75)	0.21 (−1.66, 2.08)
		**−3.35 (−5.99,−0.70)**	−1.50 (−4.27, 1.27)	−0.06 (−1.77, 1.66)	−0.13 (−2.34, 2.07)	−0.09 (−2.25, 2.07)	**Meditation** **+** **yoga**	0.57 (−1.82, 2.96)	−1.13 (−3.47, 1.21)	−1.11 (−2.58, 0.36)	−1.11 (−2.77, 0.55)	−0.79 (−2.28, 0.70)	−0.34 (−1.66, 0.98)
		**−3.32 (−6.81, 0.17)**	−1.48 (−5.06, 2.11)	−0.03 (−2.87, 2.81)	−0.11 (−3.27, 3.06)	−0.06 (−3.20, 3.07)	0.03 (−3.10, 3.15)	**Dance**	−1.70 (−4.48, 1.08)	−1.68 (−3.78, 0.42)	−1.68 (−3.91, 0.56)	−1.36 (−3.47, 0.75)	−0.91 (−2.90, 1.09)
		**−3.53 (−6.97,−0.09)**	−1.68 (−5.22, 1.85)	−0.24 (−3.03, 2.55)	−0.32 (−3.43, 2.80)	−0.27 (−3.35, 2.82)	−0.18 (−3.26, 2.89)	−0.21 (−4.03, 3.62)	**Ayurvedic massage**	0.02 (−2.02, 2.06)	0.02 (−2.16, 2.21)	0.34 (−1.72, 2.40)	0.79 (−1.14, 2.73)
		**−3.57 (−5.84,−1.31)**	−1.73 (−4.14, 0.68)	−0.28 (−1.40, 0.84)	−0.36 (−2.00, 1.29)	−0.31 (−2.03, 1.41)	−0.23 (−1.98, 1.53)	−0.25 (−3.12, 2.62)	−0.04 (−2.86, 2.77)	**Yoga**	0.00 (−1.17, 1.18)	0.32 (−0.53, 1.16)	0.77 (0.13, 1.42)
		**−3.60 (−6.02,−1.17)**	−1.75 (−4.31, 0.81)	−0.31 (−1.72, 1.10)	−0.38 (−2.37, 1.60)	−0.34 (−2.26, 1.58)	−0.25 (−2.20, 1.70)	−0.28 (−3.27, 2.72)	−0.07 (−3.01, 2.87)	−0.03 (−1.51, 1.46)	**Meditation**	0.31 (−0.80, 1.43)	0.77 (−0.24, 1.78)
		**−4.10 (−6.08,−2.12)**	**−2.26 (−4.40,−0.11)**	−0.81 (−1.67, 0.05)	−0.89 (−2.53, 0.76)	−0.84 (−2.37, 0.69)	−0.76 (−2.51, 1.00)	−0.78 (−3.65, 2.09)	−0.57 (−3.39, 2.24)	−0.53 (−1.64, 0.58)	−0.50 (−1.90, 0.90)	**Other exercises**	0.46 (−0.24, 1.15)
		**−4.59 (−6.76,−2.42)**	**−2.74 (−5.06,−0.42)**	**−1.30 (−2.08,−0.51)**	−1.37 (−2.97, 0.23)	−1.33 (−2.86, 0.21)	−1.24 (−2.76, 0.28)	−1.27 (−4.00, 1.47)	−1.06 (−3.73, 1.62)	**−1.02 (−1.89,−0.14)**	−0.99 (−2.21, 0.24)	−0.48 (−1.37, 0.40)	**Usual care**

The bold values mean the differences between one MBMBT and another one were significant.

#### 3.4.2. Disability

A total of 36 studies reported of disability, including 12 interventions and 2,921 participants. The results of the NMA showed that Usual Care was worse than Craniosacral Therapy [SMD = 4.47, 95% CI = (2.81–6.13)] and Other Manipulation [SMD = 3.34, 95% CI = (1.51–5.17)] in improving disability. The details are shown in Table 3A. There were no significant inconsistencies in any of the comparisons in the results of the node-splitting method (*P* > 0.05), thus suggesting good agreement between direct and indirect comparisons for all the studies. The details are shown in Supplementary Table 2B. The results of the SUCRA ranking of different interventions in terms of improving disability were Craniosacral Therapy (99.5%) > Other Manipulation (90.8%) > Pilates (71.2%) > Yoga (57.7%) > Meditation (56.1%) > Ayurvedic Massage (53.6%) > Other Exercises (43.7%) > Tai Chi (36.8%) > Qigong (36.6%) > Usual Care (24.0%) > Meditation + Yoga (18.6%) > Dance (11.3%). The details of this ranking are shown in Figure 5B.

#### 3.4.3. Physical health

A total of 15 studies reported on physical health, including nine interventions and 1,269 participants. The results of the NMA showed that compared with Usual Care, only cranio-sacral therapy [SMD = 4.27, 95% CI = (2.70–5.84)] was better than Usual Care in improving physical health. The details of this analysis are shown in Table 3B. There were no significant inconsistencies in any of the comparisons in the results of the node-splitting method (*P* > 0.05), thus suggesting good agreement between direct and indirect comparisons for all of the studies. The details are shown in Supplementary Table 2C. The results of the SUCRA ranking of different interventions in terms of improving disability were Craniosacral Therapy (100.0%) > Pilates (72.3%) > Meditation (55.9%) > Other Manipulation (52.9%) > Other Exercises (50.5%) > Qigong (48.6%) > Ayurvedic Massage (37.7%) > Yoga (18.0%) > Usual Care (14.2%). The details of this ranking are shown in Figure 5C.

**Table 3B:** League table on physical health and mental health.

	Mental health									
Physical health	**Craniosacral Therapy**	**−2.92 (−4.05,−1.79)**	**−2.79 (−3.97,−1.61)**	**−3.09 (−4.28,−1.90)**	**−3.03 (−3.98,−2.08)**	**−3.30 (−4.50,−2.10)**	**−3.38 (−4.89,−1.88)**	**−3.36 (−4.49,−2.23)**	**−3.62 (−4.70,−2.54)**
		**3.03 (1.45, 4.61)**	**Pilates**	0.13 (−0.70, 0.96)	−0.17 (−1.40, 1.07)	−0.11 (−0.71, 0.49)	−0.38 (−1.29, 0.53)	−0.47 (−1.68, 0.75)	−0.44 (−1.20, 0.32)	**−0.70 (−1.31,−0.09)**
		**3.40 (1.67, 5.12)**	0.37 (−0.88, 1.62)	**Meditation**	−0.30 (−1.58, 0.98)	−0.24 (−0.94, 0.46)	−0.51 (−1.47, 0.45)	−0.60 (−1.82, 0.63)	−0.57 (−1.37, 0.23)	**−0.83 (−1.47,−0.19)**
		**3.40 (1.72, 5.09)**	0.37 (−1.43, 2.18)	0.01 (−1.93, 1.94)	**Other manipulation**	0.06 (−1.02, 1.14)	−0.21 (−1.51, 1.09)	−0.30 (−1.88, 1.29)	−0.27 (−1.51, 0.97)	−0.53 (−1.72, 0.66)
		**3.50 (2.16, 4.84)**	0.47 (−0.36, 1.31)	0.11 (−0.99, 1.20)	0.10 (−1.50, 1.70)	**Other exercises**	−0.27 (−1.01, 0.47)	−0.36 (−1.52, 0.81)	−0.33 (−0.94, 0.28)	**−0.59 (−1.11,−0.07)**
		**3.54 (1.73, 5.35)**	0.51 (−0.91, 1.93)	0.14 (−1.40, 1.69)	0.14 (−1.87, 2.15)	0.04 (−1.18, 1.25)	**Qigong**	−0.09 (−1.40, 1.23)	−0.06 (−0.80, 0.68)	−0.32 (−1.12, 0.48)
		**3.84 (1.58, 6.10)**	0.81 (−1.07, 2.69)	0.44 (−1.47, 2.36)	0.44 (−1.99, 2.87)	0.34 (−1.49, 2.17)	0.30 (−1.79, 2.40)	**Ayurvedic massage**	0.02 (−1.16, 1.21)	−0.23 (−1.28, 0.81)
		**4.21 (2.55, 5.88)**	1.19 (0.02, 2.35)	0.82 (−0.45, 2.08)	0.81 (−1.07, 2.70)	0.71 (−0.28, 1.71)	0.68 (−0.54, 1.90)	0.37 (−1.48, 2.23)	**Yoga**	−0.26 (−0.81, 0.29)
		**4.27 (2.70, 5.84)**	1.24 (0.32, 2.17)	0.88 (−0.12, 1.87)	0.87 (−0.93, 2.67)	0.77 (−0.05, 1.59)	0.74 (−0.57, 2.04)	0.43 (−1.20, 2.07)	0.06 (−0.82, 0.94)	**Usual care**

The bold values mean the differences between one MBMBT and another one were significant.

#### 3.4.4. Mental health

A total of 14 studies reported of physical health, including nine interventions and 1,216 participants. The results of the NMA showed that Usual Care was worse in improving mental health than the following treatments: Craniosacral Therapy [SMD = −3.62, 95% CI = (−4.70 to −2.54)], Pilates [SMD = −0.70, 95% CI = (−1.31 to −0.09)], Meditation [SMD = −0.83, 95% CI = (−1.47 to −0.19)], and Other Exercises [SMD = −0.59, 95% CI = (−1.11 to −0.07)]. The details are shown in Table 3B. There were no significant inconsistencies in any of the comparisons in the results of the node-splitting method (*P* > 0.05), thus suggesting good agreement between direct and indirect comparisons for all of the studies. The details are shown in Supplementary Table 2D. The results of the SUCRA ranking of different interventions in terms of improving disability were Craniosacral Therapy (100.0%) > Meditation (70.7%) > Pilates (63.2%) > Other Exercises (55.9%) > Other Manipulation (48.9%) > Qigong (36.0%) > Ayurvedic Massage (32.5%) > Yoga (31.0%) > Usual Care (11.8%). The details of this ranking are shown in Figure 5D.

### 3.5. Publication bias test

**Figure 6:**
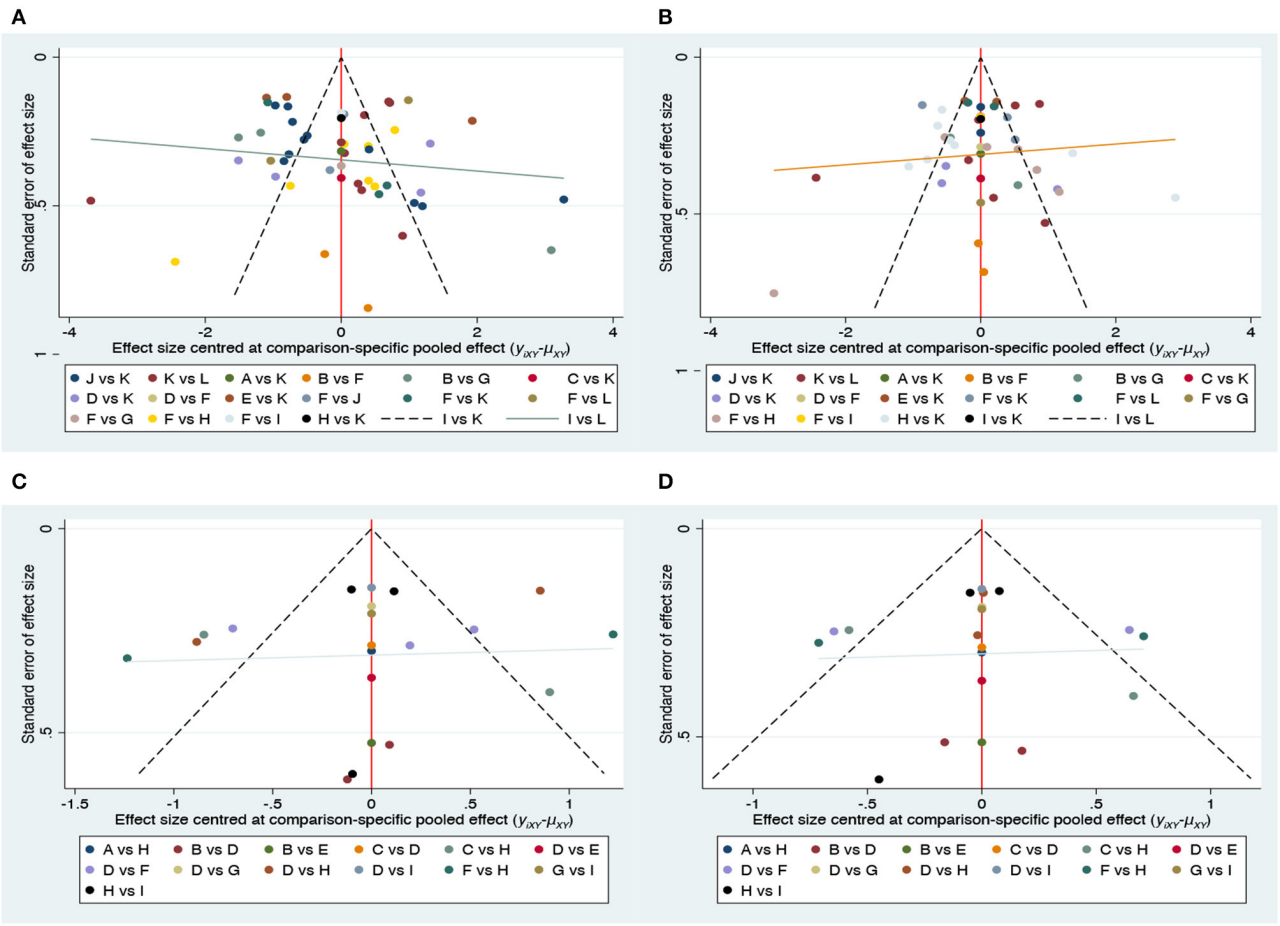
Funnel plot on publication bias. **(A)** Pain; **(B)** disability; **(C)** physical health; **(D)** mental health.

Funnel plots for all of the outcome indicators showed that most studies were evenly distributed on both sides of the red centerline, thus suggesting that publication bias is less likely to exist in this review. Details are shown in Figure 6.

## 4. Discussion

To identify the best model of MBMBT for NLBP patients, this NMA is the first to compare the effectiveness of nine different therapeutic interventions in improving patients' pain, disability, physical health, and mental health based on RCTs of 3,886 NLBP patients. This NMA showed that in terms of improving pain and disability, Craniosacral Therapy was the most effective intervention, followed by Other Manipulation and Pilates. In terms of improving physical health, Craniosacral Therapy was the most effective intervention, followed by Pilates and Meditation. In terms of improving mental health, Craniosacral Therapy was the most effective intervention, followed by Meditation and Pilates.

NLBP often includes diseases such as lumbar strain, third lumbar transverse synovial syndrome, lumbar dorsal myofasciitis, and myofibrillar tissue inflammation, with low back pain as the main symptom; in addition, the pain sites are mainly concentrated in the lower lumbar spine and lumbosacral region. NLBP patients have dysfunction of the sensorimotor system that controls muscle strength and coordination due to local pain, which reduces the activation ability and coordination of the lumbar muscles and makes them unable to withstand the daily load, which largely affects the functional activities and quality of life of patients (Pourahmadi et al., 2020). In addition, the pain produced by NLBP is not only related to physiological factors but also closely related to psychological factors. In 2020, the International Association for the Study of Pain updated the definition of pain as (Raja et al., 2020) “Pain is a distressing experience associated with actual or potential tissue damage with sensory, emotional, cognitive, and social components”, thus emphasizing the importance of the patient's subjective feelings and reinforcing the importance of the mental and psychological dimensions. This scenario indicates that physicians should consider the patient's symptoms, signs, and psychological status during treatment. This effect was also confirmed by Dinakar and Stillman (2016), who stated that pain is first transmitted to the spinal cord through afferent fibers and subsequently moves up to the thalamus for processing and integration, after which the information projects to various brain areas involved in perception, cognition and emotional components in the cerebral cortex, which elicits unpleasant emotions in patients. The patient's negative emotions are then consciously perceived, regulated, and transformed, thus resulting in an increase in the body's pain sensation and a vicious cycle of “pain-negative emotions” (Cosci et al., 2021). Therefore, in this NMA, four indicators (pain, disability, physical health, and mental health) were selected to evaluate the effects of different MBMBTs on NLBP patients.

The new finding of this NMA is that Craniosacral Therapy is the most effective MBMBT for NLBP. Cerebrospinal fluid circulates between the brain and spinal cord, which not only protects the spinal cord by cushioning vibrations but also removes metabolites and inflammatory exudates from the spinal cord and provides adequate nutrition, which is closely related to the treatment of CLBP (Laura et al., 2020). Levy (1999) applied MRI techniques to cerebrospinal fluid circulation and conducted a kinetic study; they found that the cerebrospinal fluid flow rate was slower in older individuals, thus indirectly suggesting that the cerebrospinal fluid flow rate is lower in patients with spinal degeneration than in normal subjects. Therefore, cerebrospinal fluid circulation may be the key to improving the condition of patients with NLBP. Craniosacral therapy, which was created by John E. Upledger in the United States in the 20th century, is a manipulation that loosens muscles and improves microcirculation and neurological dysfunction in the body by gently touching different parts of the entire mesolimbic system of the body from the cranial to the pelvic-sacral regions, thus adjusting the craniosacral rhythm, improving the flow of cerebrospinal fluid, and restoring normal connections and natural movements between the central nervous system and other systems of body therapy (Upledger, 1995). In addition, as a type of MBMBT, Craniosacral therapy focuses more on the interplay between brain, mind, body, and behavior, wherein patients in a relaxed state use the body's natural healing power and wisdom by noticing changes in the body in the present moment to integrate mind and body and to reregulate the body's balance while also promoting physical, emotional, and spiritual wellbeing (Haller et al., 2019). Consistent with the results of a previously published meta-analysis, Craniosacral Therapy significantly improved pain intensity, disability function, and physical and psychological quality of life in patients with chronic pain containing NLBP compared to conventional treatment (Haller et al., 2019). The multifidus is an important paravertebral muscle group that plays a key role in maintaining lumbar spine stability and is correspondingly closely related to the treatment of NLBP (James et al., 2022). Bialoszewski et al. (2014) compared the effects of Craniosacral Therapy with Other Manipulations in patients with NLBP and found that Craniosacral Therapy could differentially reduce pain intensity and frequency in patients with NLBP by decreasing the resting tension of the multifidus. In addition, the advantage of Craniosacral Therapy over other MBMBT is that it is gentle, applicable to any age group, and can stimulate the patient's potential to obtain a lasting and stable treatment effect.

The results of this NMA also showed that Pilates may be the MBMBT with the closest effectiveness to Craniosacral Therapy in the treatment of patients with NLBP. Consistent with the results of previous studies by other researchers (Fernández-Rodríguez et al., 2022; Shi et al., 2022), both demonstrate the unparalleled advantages of Pilates in the treatment of NLBP, which not only affects the stability of the lumbar spine but also causes impairment in the body's control of movement. Even if the patient's low back pain symptoms disappear and the ability to perform normal activities is restored, the muscle groups that have significant control over the stability of the human lumbar spine are difficult to restore. Therefore, in the treatment of NLBP, exercise intervention to strengthen the control of the core muscles and to restore the protective mechanism of the posterior lumbar spine to its normal biological structure is considered an effective approach. Pilates, which was also created in the 20th century, is an exercise that works with proper breathing methods to soothe the muscles of the whole body and to improve the control of the human trunk, thus aiming to improve the strength, flexibility, and posture of the body, as well as to enhance mental awareness and achieve physical and mental balance (Geneen et al., 2017). Several studies have demonstrated (Cruz-Díaz et al., 2018; Öner Suata and Karagün, 2022) that Pilates can activate deep paravertebral muscle groups, improve muscle strength and fatigue resistance, effectively improve patients' lumbar pain and dysfunction, help reduce negative emotions (such as anxiety and depression), relieve stress, enhance psychological tolerance, and improve quality of life.

All MBMBT for NLBP (a total of nine) were included in this NMA, in addition to Craniosacral Therapy and Pilates, Meditation, Yoga, Ayurvedic Massage, Yoga + Meditation, Qigong, Tai Chi, and Dance. Among them, Craniosacral Therapy and Ayurvedic Massage belong to the category of manipulation, whereas Pilates, Meditation, Yoga, Yoga + Meditation, Qigong, Tai Chi, and Dance belong to the category of movement. Ayurvedic massage and cranio-sacral therapy both have therapeutic effects by improving cerebrospinal fluid circulation and stimulating Aδ and C nerve fibers (Kumar et al., 2017; Haller et al., 2019). However, Ayurvedic massage was significantly less effective than Craniosacral Therapy, and the reason for this analysis may be because only one study on Ayurvedic massage was retrieved and could not be compared with other similar studies. Similar to the results of Park et al. (2020), Yoga, Tai Chi, and Qigong were effective in improving low back pain, dysfunction, physical health, and mental health; however, they fell into the mid-range in terms of their effectiveness in treating NLBP compared to the other therapies in this NMA. The reason for this result may be that all three therapies are a set of exercises possessing complex movements, which are initially difficult to independently complete without the leadership of a teacher. Although Meditation is more effective in terms of physical and mental health, it is less effective in terms of pain and disability. A review of previous studies found (Michalsen et al., 2016) that Meditation improves the physical and mental health of patients with NLBP; however, its efficacy is not significant because patients are plagued by long intervention times and slow results, thus resulting in poor compliance. Meditation + Yoga is a combined therapy that is supposed to be more effective than Meditation alone or Yoga alone. However, this is not the case, as Meditation or Yoga alone has better results for disability than the combination of the two. The reason for this effect may be due to the difficulty of the combined therapy and the excessive time that is needed, thus resulting in a poorer quality of movement for the patient. Dance exhibits the worst results in terms of disability, which may be due to the fact that there are fewer articles on Dance therapy NLBP, in addition to the complexity of Dance movements, which makes it difficult for most people to master its techniques in a short period of time (even with the guidance of a teacher).

Overall, this NMA provides further evidence for the effectiveness of MBMBT in the treatment of NLBP, which has certain clinical implications. First, the findings demonstrate that Craniosacral Therapy is significantly effective in improving pain, disability, physical health, and mental health in NLBP and may be the most effective MBMBT for treating patients with NLBP. In addition, Pilates is also one of the recommended treatments. Second, clinicians may consider and promote MBMBT as a good non-pharmacological treatment for NLBP management. Finally, the standardization of MBMBT application should also be guaranteed in the future; in addition, basic research on MBMBT may be considered to explore its mechanism of action.

## 5. Strengths and limitations

First, this NMA is the first analysis to compare the effectiveness of different MBMBTs for NLBP, thus identifying an optimal measure to improve pain, disability, physical health, and mental health with scientific value, as well as providing reliable evidence for the treatment of NLBP patients. No language restrictions were made during the search, and no specific interventions were prescribed. A total of 46 RCTs and 3,886 patients were involved in the analysis. Moreover, the study results were reported with authenticity and completeness. Therefore, this NMA provides comprehensive and rigorous evidence-based recommendations for managing NLBP patients.

However, there were some limitations to this NMA. As it was not possible to obtain sufficient data for individual patients in the RCTs, the analysis could only be performed at the general level; therefore, confounding factors could not be eliminated. However, we considered the particular individuals that were mentioned in each RCT; thus, these factors had little impact on the results of the NMA. In addition, the insufficient number of studies and small sample sizes that were included in some interventions may have led to the probability of false-positive results for comparison. Therefore, until more high-quality RCTs are included, readers should view these results with caution.

## 6. Conclusions

This NMA shows that Craniosacral Therapy may be the best MBMBT for treating patients with NLBP and deserves clinical promotion. When multiple MBMBTs are simultaneously applied, the results may be counterproductive and are subsequently not recommended. However, limited by the number and quality of the included studies, as well as the small number of studies with direct comparisons between MBMBT, the results may be subject to some error, and more large samples and high-quality RCTs are needed for further validation to ensure the scientific validity of the findings.

## Data availability statement

The original contributions presented in the study are included in the article/Supplementary material, further inquiries can be directed to the corresponding author.

## Author contributions

HY and XiW wrote the manuscript. WZ, YD, TS, and WC conducted the literature search, screening, data extraction, and analysis. XiW adjudicated all disagreements and provided technical guidance. XuW and JY contributed to the design and revision of the manuscript. WW contributed to the revision of the manuscript. All authors contributed to this paper, read the full text, and agreed to send the manuscript.
