# Supplementary material for: Effect of mindfulness-based mind-body therapies in patients with non-specific low back pain—A network meta-analysis of randomized controlled trials
Source: Front Aging Neurosci. 2023 Jun 29;15:1148048. doi: 10.3389/fnagi.2023.1148048 (PMC10340124; doi:10.3389/fnagi.2023.1148048)
Supplement: Supplementary file 1 [file Data_Sheet_1.pdf]

frontiers

Supplementary Material

Effect of Mindfulness-Based Mind-Body Therapies in Patients with Non-Specific Low Back Pain-A Network Meta-Analysis of Randomized Controlled Trials

Huanying Yang, Xiangfu Wang†*, Xuetao Wang1, Jianxia Yang[1], Wanqian Zhang, Yanfang Ding, Tingrui Sang, Weiguo Chen and Wanhong Wang[3]

*** Correspondence:** Xiangfu Wang: wangxf_1969@163.com

1 Supplementary Data

1.1 Supplementary 1. Search strategy for each database

Database: PubMed <inception to January 1 2023> **（1000）**

#1	(((((((((((((((((((((((biofeedback, psychology) OR (beurofeedback)) OR (breathing exercises)) OR (qigong)) OR (aromatherapy)) OR (laughter therapy)) OR ((imagery, psychotherapy)) OR (meditation)) OR (mental Healing)) OR (psychodrama)) OR (role playing)) OR (hypnosis)) OR (autogenic training)) OR (suggestion)) OR (psychophysiology)) OR (tai chi)) OR (relaxation therapy)) OR (therapeutic touch)) OR (yoga)) OR (dance therapy)) OR (pilates)) OR (baduanjin)) OR (Mind-Body Therapies)) AND ((((nonspecific low back pain) OR (non-specific low back pain)) OR (NSLBP)) OR (NLBP))

Database: EMBASE <inception to January 1 2023> **（273）**

#1	nonspecific AND low AND back AND pain OR ('non specific' AND low AND back AND pain) OR nslbp OR nlbp
#2	'mind body' AND therapies
#3	biofeedback, AND psychology OR beurofeedback OR (breathing AND exercises) OR qigong OR aromatherapy OR (laughter AND therapy) OR (imagery, AND psychotherapy) OR meditation OR (mental AND healing) OR psychodrama OR (role AND playing) OR hypnosis OR (autogenic AND trainin) OR suggestion OR psychophysiology OR (tai AND chi) OR (relaxation AND therapy) OR (therapeutic AND touch) OR yoga OR (dance AND therapy) OR pilates OR baduanjin

#4	#2 OR #3
#5	#1 AND #4

Database: Cochrane Central Register of Controlled Trials <inception to January 1 2023> **（342）**

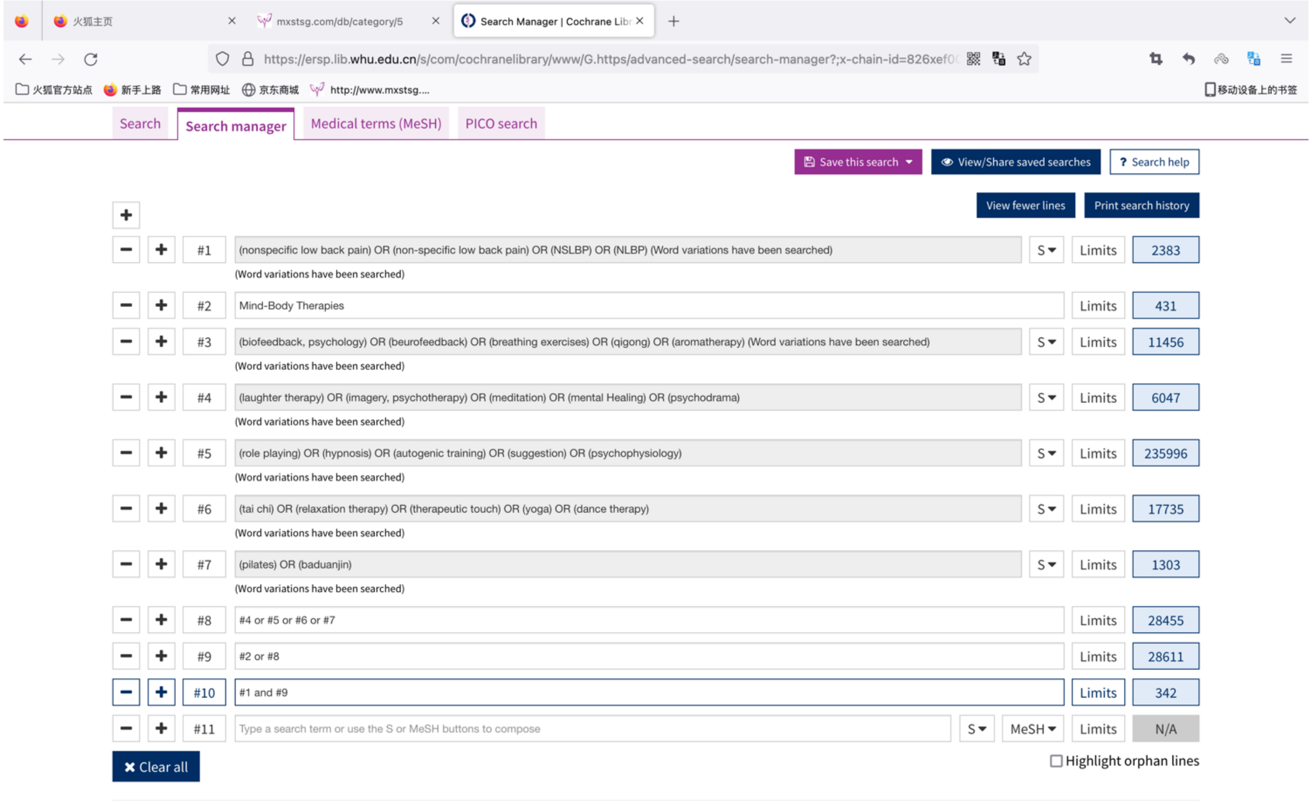

Database: Web of Science <inception to January 1 2023> **（1522）**

#1	((((ALL=(nonspecific low back pain)) OR ALL=(non-specific low back pain)) OR ALL=(NSLBP)) OR ALL=(NLBP)) OR ALL=(Mind-Body Therapies)
#2	((((((((((((((((((ALL=(biofeedback, psychology)) OR ALL=(beurofeedback)) OR ALL=(breathing exercises)) OR ALL=(qigong)) OR ALL=(aromatherapy)) OR ALL=(laughter therapy)) OR ALL=(imagery, psychotherapy)) OR ALL=(meditation)) OR ALL=(mental Healing)) OR ALL=(psychodrama)) OR ALL=(role playing)) OR ALL=(hypnosis)) OR ALL=(autogenic training)) OR ALL=(suggestion)) OR ALL=(psychophysiology)) OR ALL=(tai chi)) OR ALL=(relaxation therapy)) OR ALL=(therapeutic touch)) OR ALL=(yoga)) OR ALL=(dance therapy)) OR ALL=(pilates)) OR ALL=(baduanjin)

#3	#1 AND #2

1.2 Supplementary 2A. Consistency test for pain.

Side	Direct		Indirect		Difference			tau
	Coef.	Std. Err.	Coef.	Std. Err.	Coef.	Std. Err.	P>z	
A K *	1.057527	1.37093	1.097804	15.25325	-0.04028	15.31473	0.998	1.333896
B F *	5.195197	0.999289	-4.01454	2.815622	9.209735	3.038694	0.002	1.196101
B G *	1.630056	0.799905	6.474052	3.968202	-4.844	4.07112	0.234	1.319988
C K *	1.266217	1.394239	0.849407	200.0368	0.41681	200.0425	0.998	1.333728
D F	-0.28116	1.375544	0.801342	0.845213	-1.0825	1.614468	0.503	1.345375
D K	1.191887	0.698663	0.109658	1.455264	1.08223	1.614292	0.503	1.34537
E K *	1.240502	0.775929	0.874995	115.5095	0.365508	115.5121	0.997	1.333736
F G	-0.13305	1.273168	-5.8085	1.659501	5.67545	2.090351	0.007	1.219591
F H	-1.09321	0.529331	-0.1926	0.78452	-0.90061	0.946482	0.341	1.337315
F I	0.212923	1.344925	-1.58675	1.070762	1.799675	1.719114	0.295	1.331739
F J	-0.78561	0.981314	-0.9517	1.341757	0.166092	1.659205	0.92	1.355392
F K	1.115145	0.801663	0.189515	0.548145	0.92563	0.971422	0.341	1.338398
F L	-1.11393	0.968069	-0.22414	0.702063	-0.88979	1.195813	0.457	1.34331
H K	1.108833	0.46073	1.926063	0.844518	-0.81723	0.961799	0.395	1.340999
I K	1.887292	1.380559	1.087217	1.0302	0.800075	1.722573	0.642	1.350521
I L	0.960559	1.364128	-0.02089	1.081412	0.981449	1.740776	0.573	1.348684
J K	1.607145	0.988439	0.818139	1.336042	0.789005	1.664395	0.635	1.351338
K L	-0.88758	0.494061	-1.64348	1.095081	0.7559	1.20137	0.529	1.346631

1.3 Supplementary 2B. Consistency test for disability.

Side	Direct		Indirect		Difference			tau
	Coef.	Std. Err.	Coef.	Std. Err.	Coef.	Std. Err.	P>z	
A K *	0.793531	0.990168	0.756588	16.90445	0.036943	16.93342	0.998	0.941096
B F *	4.184427	0.810183	2.14112	2.645407	2.043307	2.757549	0.459	0.951104
B G *	1.088105	0.717582	1.841983	3.04118	-0.75388	3.145547	0.811	0.957151
C K *	-0.90611	1.017401	2.493282	200.0092	-3.3994	200.0125	0.986	0.94102
D F	0.227913	1.005302	0.360335	0.711298	-0.13242	1.231494	0.914	0.963759
D K	0.801661	0.600355	0.669257	1.075147	0.132404	1.23144	0.914	0.963757
E K *	-0.33706	0.672763	1.924364	141.432	-2.26143	141.4337	0.987	0.941022
F G	-2.50342	1.056036	-3.67641	1.522844	1.17299	1.846365	0.525	0.951839
F H	-0.88017	0.42234	-0.21757	0.62388	-0.6626	0.753238	0.379	0.949749
F I	0.480188	0.98058	-0.00777	0.797064	0.487958	1.263665	0.699	0.961974
F K	0.520776	0.569566	0.408224	0.471502	0.112553	0.739954	0.879	0.964654
F L	-0.05076	0.686985	-0.50568	0.57269	0.454922	0.894372	0.611	0.959745
H K	1.067441	0.356867	1.360005	0.693581	-0.29256	0.779432	0.707	0.962716
I K	0.580797	0.99099	0.089892	0.747083	0.490906	1.241046	0.692	0.961248

I L	-0.518	0.984931	-0.49792	0.789106	-0.02007	1.262053	0.987	0.965085
J K *	0.211657	0.954287	1.375314	200.0075	-1.16366	200.0099	0.995	0.94102
K L	-0.78088	0.358966	-0.71649	0.99975	-0.06439	1.062642	0.952	0.964823

1.4 Supplementary 2C. Consistency test for physical health.

Side	Direct		Indirect		Difference			tau
	Coef.	Std. Err.	Coef.	Std. Err.	Coef.	Std. Err.	P>z	
A H *	-0.43173	0.833965	-1.18744	26.72694	0.755709	26.73995	0.977	0.778219
B D *	-3.50149	0.682737	4.180309	141.4284	-7.6818	141.4312	0.957	0.778182
B E *	-3.3431	0.967039	-3.76886	2.584481	0.425762	2.812322	0.88	0.812082
C D	0.211776	0.858766	-0.36178	0.781387	0.573555	1.161053	0.621	0.809786
C H	-1.03826	0.619725	-0.46465	0.981457	-0.57361	1.161017	0.621	0.809783
D E *	0.05738	0.89042	0.481605	2.665671	-0.42423	2.812207	0.88	0.812079
D F	0.567996	0.495272	0.079499	1.013154	0.488497	1.126891	0.665	0.817568
D G	-0.46018	0.813324	0.60674	1.00115	-1.06692	1.289883	0.408	0.790847
D H	-0.68645	0.602848	-0.87159	0.642265	0.18514	0.880294	0.833	0.822747
D I	0.053659	0.759377	-1.25273	0.639184	1.306391	0.992598	0.188	0.745506
F H	-1.4406	0.609823	-0.88203	0.83582	-0.55857	1.03464	0.589	0.812261
G I	-1.10697	0.817813	-0.03996	0.997491	-1.06702	1.289887	0.408	0.790847
H I	0.003691	0.512155	0.351471	1.198544	-0.34778	1.303579	0.79	0.82378

1.5 Supplementary 2D. Consistency test for mental health.

Side	Direct		Indirect		Difference			tau
	Coef.	Std. Err.	Coef.	Std. Err.	Coef.	Std. Err.	P>z	
A H *	-0.234201	0.534511	-0.9911	27.73262	0.756898	27.73777	0.978	0.444164
B D *	-3.02857	0.485228	3.740344	141.4421	-6.76891	141.4439	0.962	0.444155
B E *	-3.193695	0.691784	-2.51771	1.781821	-0.67598	1.978198	0.733	0.464023
C D	-0.047259	0.549567	-0.39648	0.504245	0.349224	0.745847	0.64	0.46984
C H	-0.928314	0.404515	-0.57905	0.626809	-0.34927	0.745836	0.64	0.469839
D E *	9.85E-10	0.590465	-0.67664	1.887993	0.676635	1.978172	0.732	0.464022
D F	0.2629638	0.365669	-0.32089	0.61258	0.583851	0.712553	0.413	0.455459
D G	-0.214295	0.523466	-0.36022	0.64861	0.145921	0.833493	0.861	0.488589
D H	-0.594044	0.374015	-0.58084	0.435831	-0.0132	0.574684	0.982	0.486496
D I	-0.231735	0.508456	-0.41581	0.451404	0.184075	0.679961	0.787	0.4874
F H	-0.815877	0.384754	-0.38879	0.628916	-0.42709	0.737909	0.563	0.474546
G I	-0.004978	0.525457	-0.15089	0.646999	0.145908	0.833495	0.861	0.488589
H I	0.2180391	0.326738	0.447633	0.757729	-0.22959	0.825392	0.781	0.484974
